# Association of the *PSRC1* rs599839 Variant with Coronary Artery Disease in a Mexican Population

**DOI:** 10.3390/medicina56090427

**Published:** 2020-08-26

**Authors:** Martha Eunice Rodríguez-Arellano, Jacqueline Solares-Tlapechco, Paula Costa-Urrutia, Helios Cárdenas-Hernández, Marajael Vallejo-Gómez, Julio Granados, Sergio Salas-Padilla

**Affiliations:** 1 Laboratorio de Medicina Genómica, Hospital Regional Lic. Adolfo López Mateos ISSSTE, Ciudad de Mexico 01030, Mexico; marthaeunicer@yahoo.com.mx (M.E.R.-A.); jsoltlapechco@gmail.com (J.S.-T.); paula.costa.urrutia@gmail.com (P.C.-U.); ibi_cardenashelios@yahoo.com.mx (H.C.-H.); marajael_vg@hotmail.com (M.V.-G.); 2 División de Inmunogenética, Departamento de Trasplantes, Instituto Nacional de Ciencias Médicas y Nutrición Salvador Zubirán, Ciudad de Mexico 14080, Mexico; julgrate@yahoo.com; 3 Servicio de Cardiología, Hospital Regional Lic. Adolfo López Mateos ISSSTE, Ciudad de Mexico 01030, Mexico

**Keywords:** coronary artery disease, ischemic heart disease, cardiovascular diseases, coronary heart disease, SNP

## Abstract

*Background and Objectives:* Coronary artery disease (CAD) is a major health problem in México. The identification of modifiable risk factors and genetic biomarkers is crucial for an integrative and personalized CAD risk evaluation. In this work, we aimed to validate in a Mexican population a set of eight selected polymorphisms previously associated with CAD, myocardial infarction (MI), or dyslipidemia. *Materials and Methods*: A sample of 907 subjects (394 CAD cases and 513 controls) 40–80 years old was genotyped for eight loci: *PSRC1* (rs599839), *MRAS* (rs9818870), *BTN2A1* (rs6929846), *MTHFD1L* (rs6922269), *CDKN2B* (rs1333049), *KIAA1462* (rs3739998), *CXCL12* (rs501120), and *HNF1A* (rs2259816). The association between single nucleotide polymorphisms (SNPs) and CAD was evaluated by logistic regression models. *Results:* Multiple logistic regression analysis with adjustment by age, gender, and body mass index showed that rs599839 was significantly associated with CAD (OR_ADD_ = 0.72, *p* = 0.009; OR_DOM_ = 0.66, *p* = 0.007). *Conclusions:* The *PSRC1* rs599839 polymorphism shows a significant protective association with CAD in this sample of the Mexican population.

## 1. Introduction

Noncommunicable diseases (NCDs) are the world’s leading cause of death. In 2016, an estimate of 41 million deaths (71% out of the total of 51 million deaths) were due to NCDs, and 44% of them were attributable to cardiovascular disease (CVD) [1], including coronary heart disease (CHD), also named coronary artery disease (CAD), and ischemic heart disease (IHD) [2,3]. In 1990, CAD was the second leading cause of death in developing countries and currently ranks first [4].

In Mexico, the proportion of deaths attributable to CVD has increased from 22% in 2000 to 26% in 2018; 57% of those deaths were due to CAD [5]. The clinical manifestations of CAD include acute myocardial ischemia, unstable angina (UA), non–ST-segment elevation myocardial infarction (NSTEMI), and ST-segment elevation myocardial infarction (STEMI) [6]. CAD has a complex etiology involving environmental risk factors and genetic susceptibility; this fact highlights the importance of finding genetic markers for early risk assessment. During the last years, the search for CVD predictors has focused on the development of genetic risk scores [7,8] by means of robust and replicable genome-wide studies in extremely large cohorts that have contributed to identifying several genetic loci and their association with CAD, myocardial infarction, and dyslipidemia in Europeans and Asians [9,10,11,12,13,14,15,16].

Strategies aimed to reduce cardiovascular risk factors such as abnormal lipids, smoking, hypertension, diabetes, and abdominal obesity [17] are currently used to deal with the burden of CAD; however, genetic risk has not been widely explored in the Mexican population yet. The present study aimed to assess the genetic susceptibility of Mexicans to CAD through the evaluation of eight CAD-associated loci previously identified by GWAS in Europeans and Asians.

## 2. Materials and Methods

### 2.1. Study Design

We performed a case-control study with subjects meeting the following criteria: men or women 40–80 years old, Mexican-Mestizo ancestry (parents and grandparents born in Mexico), not related to other study participants, and diagnosis of CAD to be considered as cases, or no previous record of CAD or known cardiovascular disease to be included in the control group.

All cases were recruited at the Cardiology Service of Hospital Regional Lic. Adolfo López Mateos, ISSSTE (Instituto de Seguridad y Servicios Sociales de los Trabajadores del Estado) and controls at the Automated Diagnosis and Detection Clinic (Clínica de Detección y Diagnóstico Automatizado, CLIDDA, ISSSTE) in Mexico City. All subjects gave their informed consent for inclusion before they participated in the study. The study was conducted in accordance with the Declaration of Helsinki, and the protocol was approved by the Ethics and Biosafety Committees of the Regional Hospital Lic. Adolfo López Mateos (registry number 246.2012).

### 2.2. Clinical, Anthropometric, and Biochemical Evaluation

For clinical evaluation, volunteers were recruited as cases if diagnosed with stable or unstable angina, myocardial ischemia, or myocardial infarction. Stable angina was diagnosed by the presence of precordial pain at effort that subsided at rest, with no electrocardiographic alterations, and biochemical markers (creatine phosphokinase [CPK] and CPK-MB) at the normal range. Unstable angina was characterized by prolonged precordial pain at rest with or without electrocardiographic alterations and increased biochemical markers. Myocardial ischemia was considered when precordial pain was present at rest or after >20 min exercise with or without changes in the basal ECG at rest, and with or without increased enzymatic markers. Myocardial infarction was diagnosed by the presence of precordial pain, ECG with or without ST-segment elevation in subcategory of CAD 2 or more contiguous derivations of 2 mm in V1-3 and 1 mm in all other derivations, and increased biochemical markers.

Anthropometric measurements of weight and height were obtained to calculate the body mass index (BMI) as weight (kg) divided by squared height (m).

Biochemical parameters were evaluated in all participants. Glucose, total cholesterol, triglycerides (TG), high-density lipoprotein cholesterol (HDLc), low-density lipoprotein cholesterol (LDLc) (in mg/dL), and the percentage of glycated hemoglobin (HbA1c in %) were routinely determined for all subjects after 10 h fasting.

### 2.3. Marker Selection and Genotyping

We selected a set of eight common variants (Table 1) previously associated with CAD, MI, or dyslipidemia, and with genome-wide significance (*p* < 5 × 10^−8^) either in a single study or in a meta-analysis.

Genomic DNA was obtained from 200 μL of whole blood-EDTA with the InviMag Blood DNA mini kit using an automated platform for nucleic acid isolation (InviGenius, Stratec; Berlin, Germany). Selected SNPs were genotyped by using a predesigned 5′ exonuclease TaqMan genotyping assay on a 7500 series Real-Time PCR system, according to the manufacturer’s instructions (Applied Biosystems, Foster City, CA, USA).

### 2.4. Statistical Analysis

General characteristics of cases and controls were compared by the Student’s *t*-test using STATA/SE12.0 (StataCorp College Station, TX, USA). The Hardy–Weinberg equilibrium was evaluated using PLINK v1.07 (http://pngu.mgh.harvard.edu/purcell/plink, Harvard University, Cambridge, MA, USA). Logistic regression models were used to test the association between each polymorphism and CAD, adjusting by sex, age, and BMI under additive, dominant, and recessive inheritance models using STATA/SE12.0. No Bonferroni adjustment was applied, since we tested each gene as an independent hypothesis and the SNPs have established associations with CAD in Europeans and Asians; thus, statistical significance was considered when *p* < 0.05.

We performed power calculations using case-control design in Quanto software version 1.2.4 (University of Southern California, Los Angeles, CA, USA; http://biostats.usc.edu/Quanto.html). Calculations were carried out for gene only, under an additive, dominant, and recessive inheritance model and using a range of allele frequency from 0.05 to 0.40. Our study had 80% statistical power to detect a protective effect OR ≤ 0.5 with an allele frequency of 0.05; as allele frequency increased to 0.15, OR = 0.7 could be detected. For risk effect, 0.05 and 0.10 of allele frequency, OR ≥ 1.8 and OR ≥ 1.4 could be detected, respectively.

## 3. Results

A sample of 907 subjects (394 CAD cases and 513 controls) was recruited for this study and genotyped for selected variants (Table 1). Subjects’ characteristics are shown in Table 2. The mean age, HbA1c, and serum TG levels were not different between controls and CAD patients (*p* > 0.05). Higher levels of serum glucose and HDLc were observed in CAD patients, while total cholesterol and LDLc were lower compared with the control group.

The results of the association between SNPs and CAD under different inheritance models are shown in Table 3. The eight SNPs were in Hardy–Weinberg equilibrium (*p* > 0.05). We found that the G allele of the rs599839 variant in the *PSRC1* gene conferred protection against CAD under additive and dominant models (OR_ADD_ = 0.72, *p* = 0.009; OR_DOM_ = 0.66, *p* = 0.007). The rest of the polymorphisms were not significantly associated with CAD.

## 4. Discussion

In this study, we evaluated the association between CAD and common SNPs, previously associated with cardiovascular disease in Europeans and Asians, in a Mexican population. A healthier profile was observed in cases compared with controls for known cardiovascular risk factors such as BMI, total cholesterol, LDLc, and HDLc due to the fact that most of the CAD patients were already under statin therapy, which acts as a competitive inhibitor of 3-hydroxymethylglutaryl coenzyme A (HMG-CoA) reductase, decreasing cholesterol biosynthesis and serum LDLc, and also triglyceride levels [19,20]. The minor G allele of the rs599839 variant in the *PSRC1* gene showed a protective effect against CAD (OR_ADD_ = 0.72, 95% CI: 0.56–0.92, *p* = 0.009; OR_DOM_ = 0.66, 95% CI: 0.49–0.89, *p* = 0.007). In line with our results, the major allele A of this variant has been reported as a risk factor for CAD (OR = 1.29, 95% CI: 1.18–1.40) in European populations [13], and similar results were observed in meta-analysis in both Europeans (OR = 1.11, 95% CI: 1.08–1.15) [11] and Asians (OR = 1.31, 95% CI: 1.17–1.47) [21]. Besides, the minor allele G has been associated with a protective effect against the risk of CAD (OR = 0.422, 95% CI: 0.181–0.981) and lower levels of total cholesterol and LDLc in Indians [22]. Also, it was found to reduce the risk of stenosis in Arab CAD patients (OR = 0.51, 95% CI: 0.30–0.92) [23], and to lower serum LDLc levels in Japanese (OR = 0.7, 95% CI: 0.57–0.85) [24]. This rs599839 polymorphism is located on the 1p13.3 chromosomal region near the *SORT1* (sortilin-1) gene, which encodes the VPS10P domain-sorting receptors involved in intracellular protein trafficking and the modulation of hepatic very-low-density protein secretion [25,26]. The frequency of the rs599839 polymorphism in the Chinese population has been reported to be higher in CAD patients compared with healthy controls (OR = 8.37, 95% CI: 1.70–41.0); in addition, CAD patients with the AA genotype display higher sortilin levels than those with the GG and GA genotypes (391.25 ± 128.24 ng/L vs. 314.70 ± 64.65 ng/L, *p* = 0.000) [27]. Other studies have also shown that the increase in SORT1 expression was negatively correlated with LDLc levels [28]. All this evidence together supports the influence of the *PSRC1* rs599839 variant in CAD genetic susceptibility in many populations.

Although no significant effect was found (*p* ≥ 0.05) for the rest of the SNPs, there are two important issues to be discussed before accepting the lack of their contribution to the CAD phenotype. First, linkage disequilibrium between SNPs and causal loci in our population could be weaker than in populations with European or Asian ancestry, leading to an association that is not easily detected. In fact, in an admixture like the Mexican population, allele frequency may change with respect to the parental population, modifying the pattern of LD, the size effect, and the direction of the association [29]. Second, if the size effect is very low in the Mexican population, it could only be detected by increasing the statistical power with a larger sample size [30].

Candidate genetic markers for risk assessment should independently be replicated in the intended specific population; in our case, of the eight variants analyzed, only one showed significant association with CAD in Mexicans.

## 5. Conclusions

We found that the *PSRC1* rs599839 polymorphism could be a protective factor against CAD; thus, it may be worth continuing to study *PSRC1* polymorphisms as potential risk-modifying genetic factors in the Mexican population.

The validation of genetic markers previously studied in other populations and associated with CAD, MI, and dyslipidemia may lead to the discovery of better markers for personalized cardiovascular risk assessment in Mexicans.

## Figures and Tables

**Table 1:** Genetic variants previously associated with CAD and selected for this study.

SNP	Gene	Chr:Position ^a^	Phenotype ^b^	Population ^c^	Reference
rs599839	*PSRC1*	1:109279544	CAD, IS	EUR	[10,11,13]
rs9818870	*MRAS*	3:138403280	CAD	EUR	[10,14]
rs6929846	*BTN2A1*	6:26458037	MI, Dyslipidemia	JAP	[15,16]
rs6922269	*MTHFD1L*	6:150931849	CAD	EUR	[13]
rs1333049	*CDKN2B*	9:22125504	CAD	EUR	[10,13,18]
rs3739998	*KIAA1462*	10:30027143	CAD, MI	EUR	[12]
rs501120	*CXCL12*	10:44258419	CAD	EUR	[13]
rs2259816	*HNF1A*	12:120997784	CAD	EUR	[14]

^a^ GRCh38.p12. ^b^ CAD, coronary artery disease; MI, myocardial infarction; IS, ischemic stroke. ^c^ JAP, Japanese; EUR, European.

**Table 2:** Characteristics of the study sample.

Characteristic	Controls (*n* = 513)	CAD (*n* = 394)	*p*-Value
Age (years)	59.7 (±9.6)	59.8 (±7.7)	0.866
BMI (kg/m^2^)	28 (±4.7)	27 (±3.8)	<0.001
Gender *n* (%)			<0.001
Male	260 (50.7%)	297 (75.3%)	
Female	253 (49.3%)	97 (24.6%)	
Glucose (mg/dL)	114.0 (±51.1)	151.2 (±90.1)	<0.001
HbA1c (%)	6.16 (± 1.46)	6.0 (±1.99)	0.270
Total cholesterol (mg/dL)	199.4 (± 51.2)	188.8 (±75.5)	0.014
Triglycerides (mg/dL)	171.8 (± 113.1)	174.4 (±91.8)	0.718
HDLc (mg/dL)	44.3 (± 14.8)	56.5 (±34.3)	<0.001
LDLc (mg/dL)	113.5 (± 47.1)	65.5 (±44.1)	<0.001

Data are presented as mean ± SD.

**Table 3:** Association analysis of selected variants.

Gen	SNP	AA	MAF	OR_ADD_ (95% CI), *p*	OR_DOM_ (95% CI), *p*	OR_REC_ (95% CI), *p*
*PSRC1*	rs599839	G	0.20	0.72 (0.56–0.92), 0.009	0.66 (0.49–0.89), 0.007	0.70 (0.35–1.38), 0.313
*MRAS*	rs9818870	T	0.05	0.86 (0.56–1.32), 0.509	0.82 (0.53–1.27), 0.383	-
*BTN2A1*	rs6929846	T	0.28	0.94 (0.75–1.18), 0.631	0.95 (0.71–1.26), 0.735	0.87 (0.51–1.47), 0.616
*MTHFD1L*	rs6922269	A	0.37	1.02 (0.84–1.25), 0.778	1.03 (0.78–1.38), 0.791	1.03 (0.70–1.53), 0.853
*CDKN2B*	rs1333049	G	0.49	0.99 (0.81–1.22), 0.997	0.91 (0.66–1.27), 0.619	1.08 (0.77–1.51), 0.619
*KIAA1462*	rs3739998	G	0.45	1.12 (0.92–1.37), 0.248	1.09 (0.80–1.48), 0.578	1.27 (0.90–1.80), 0.169
*CXCL12*	rs501120	C	0.26	0.99 (0.79–1.25), 0.985	0.93 (0.69–1.24), 0.640	1.26 (0.73–2.16), 0.402
*HNF1A*	rs2259816	T	0.41	1.22 (0.98–1.52), 0.067	1.35 (0.97–1.87), 0.066	1.24 (0.83–1.84), 0.286

Inheritance models: AA, Associated allele; MAF, Minor Allele Frequency; ADD, additive; DOM, dominant; REC, recessive.

## References

[1] World Health Organization (2018). World Health Statistics 2018: Monitoring Health for the SDGs, Sustainable Development Goals. Geneva. https://apps.who.int/iris/bitstream/handle/10665/272596/9789241565585-eng.pdf.

[2] Sanchis-Gomar F., Perez-Quilis C., Leischik R., Lucia A. (2016). Epidemiology of coronary heart disease and acute coronary syndrome. Ann. Transl. Med..

[3] Benjamin E.J., Virani S.S., Callaway C.W., Chamberlain A.M., Chang A.R., Cheng S., Chiuve S.E., Cushman M., Delling F.N., Deo R. (2018). Heart disease and stroke statistics—2018 update: A report from the American Heart Association. Circulation.

[4] Okrainec K., Banerjee D.K., Eisenberg M.J. (2004). Coronary artery disease in the developing world. Am. Heart J..

[5] Instituto Nacional de Estadística y Geografía (INEGI) Data of Méxicós general deaths from the INEGI. https://www.inegi.org.mx/sistemas/olap/Proyectos/bd/continuas/mortalidad/MortalidadGeneral.asp.

[6] Kumar A., Cannon C.P. (2009). Acute coronary syndromes: Diagnosis and management, part I. Mayo Clin. Proc..

[7] McPherson R., Tybjaerg-Hansen A. (2016). Genetics of Coronary Artery Disease. Circ. Res..

[8] Khera A.V., Kathiresan S. (2017). Genetics of coronary artery disease: Discovery, biology and clinical translation. Nat. Rev. Genet..

[9] Van der Harst P., Verweij N. (2018). Identification of 64 novel genetic loci provides an expanded view on the genetic architecture of coronary artery disease. Circ. Res..

[10] Dichgans M., Malik R., König I.R., Rosand J., Clarke R., Gretarsdottir S., Thorleifsson G., Mitchell B.D., Assimes T.L., Levi C. (2014). Shared genetic susceptibility to ischemic stroke and coronary artery disease: A genome-wide analysis of common variants. Stroke.

[11] Schunkert H., König I.R., Kathiresan S., Reilly M.P., Assimes T.L., Holm H., Preuss M., Stewart A.F.R., Barbalic M., Gieger C. (2011). Large-scale association analysis identifies 13 new susceptibility loci for coronary artery disease. Nat. Genet..

[12] Erdmann J., Willenborg C., Nahrstaedt J., Preuss M., König I.R., Baumert J., Linsel-Nitschke P., Gieger C., Tennstedt S., Belcredi P. (2011). Genome-wide association study identifies a new locus for coronary artery disease on chromosome 10p11.23. Eur. Heart J..

[13] Samani N.J., Erdmann J., Hall A.S., Hengstenberg C., Mangino M., Mayer B., Dixon R.J., Meitinger T., Braund P., Wichmann H.-E. (2007). Genomewide association analysis of coronary artery disease. N. Engl. J. Med..

[14] Erdmann J., Großhennig A., Braund P.S., König I.R., Hall A.S., Linsel-nitschke P., Kathiresan S., Wright B., Tregouet D.A., Cambien F. (2009). New susceptibility locus for coronary artery disease on chromosome 3q22.3. Nat. Genet..

[15] Fujimaki T., Kato K., Oguri M., Yohida T., Horibe H., Yokoi K., Watanabe S., Satoh K., Aoyagi Y., Tanaka M. (2011). Association of a polymorphism of BTN2A1 with dyslipidemia in East Asian populations. Exp. Ther. Med..

[16] Yamada Y., Nishida T., Ichihara S., Sawabe M., Fuku N., Nishigaki Y., Aoyagi Y., Tanaka M., Fujiwara Y., Yoshida H. (2011). Association of a polymorphism of BTN2A1 with myocardial infarction in East Asian populations. Atherosclerosis.

[17] Yusuf S., Hawken S., Ounpuu S., Dans T., Avezum A., Lanas F., McQueen M., Budaj A., Pais P., Varigos J. (2004). Effect of potentially modifiable risk factors associated with myocardial infarction in 52 countries (the INTERHEART study): Case-control study. Lancet.

[18] Preuss M., König I.R., Thompson J.R., Erdmann J., Absher D., Assimes T.L., Blankenberg S., Boerwinkle E., Chen L., Cupples A.L. (2010). Design of the Coronary ARtery DIsease Genome-Wide Replication And Meta-Analysis (CARDIoGRAM) Study: A Genome-wide association meta-analysis involving more than 22,000 cases and 60,000 controls. Circ. Cardiovasc. Genet..

[19] Sirtori C.R. (2014). The pharmacology of statins. Pharmacol Res..

[20] Oesterle A., Laufs U., Liao J.K. (2017). Pleiotropic Effects of Statins on the Cardiovascular System. Circ Res..

[21] Guo J., Luo Y.X., Tao L.X., Guo X.H. (2015). Association between 1p13.3 genomic markers and coronary artery disease: A meta-analysis involving patients and controls. Genet. Mol Res..

[22] Arvind P., Nair J., Jambunathan S., Kakkar V.V., Shanker J. (2014). CELSR2-PSRC1-SORT1 gene expression and association with coronary artery disease and plasma lipid levels in an Asian Indian cohort. J. Cardiol..

[23] Rizk N.M., El-Menyar A., Egue H., Wais I.S., Baluli H.M., Alali K., Farag F., Younes N., Al Suwaidi J. (2015). The association between serum LDL cholesterol and genetic variation in chromosomal locus 1p13.3 among coronary artery disease patients. Biomed. Res. Int..

[24] Abe S., Tokoro F., Matsuoka R., Arai M., Noda T., Watanabe S., Horibe H., Fujimaki T., Oguri M., Kato K. (2015). Association of genetic variants with dyslipidemia. Mol. Med. Rep..

[25] Carlo A.S., Nykjaer A., Willnow T.E. (2014). Sorting receptor sortilin—A culprit in cardiovascular and neurological diseases. J. Mol. Med..

[26] Musunuru K., Strong A., Frank-kamenetsky M., Lee N.E., Ahfeldt T., Sachs K.V., Li X., Li H., Kuperwasser N., Ruda V. (2010). From noncoding variant to phenotype via SORT1 at the 1p13 cholesterol locus. Nature.

[27] Han W., Wei Z., Zhang H., Geng C., Dang R., Yang M., Zhang J., Wang C., Jiang P. (2020). The association between sortilin and inflammation in patients with coronary heart disease. J. Inflamm. Res..

[28] Linsel-Nitschke P., Heeren J., Aherrahrou Z., Bruse P., Gieger C., Illig T., Prokisch H., Heim K., Doering A., Peters A. (2010). Genetic variation at chromosome 1p13.3 affects sortilin mRNA expression, cellular LDL-uptake and serum LDL levels which translates to the risk of coronary artery disease. Atherosclerosis.

[29] Pfaff C.L., Parra E.J., Bonilla C., Hiester K., McKeigue P.M., Kamboh M.I., Hutchinson R.G., Ferrell R.E., Boerwinkle E., Shriver M.D. (2001). Population structure in admixed populations: Effect of admixture dynamics on the pattern of linkage disequilibrium. Am. J. Hum. Genet..

[30] Lu Y., Loos R.J. (2013). Obesity genomics: Assessing the transferability of susceptibility loci across diverse populations. Genome Med..

